# Relationship Between Childhood Abuse and Body Mass Index in Young Adulthood: Mediated by Depression and Anxiety?

**DOI:** 10.1177/10775595221092946

**Published:** 2022-06-02

**Authors:** Leonie K. Elsenburg, Aart C. Liefbroer, Annelies E. van Eeden, Hans W. Hoek, Albertine J. Oldehinkel, Nynke Smidt

**Affiliations:** 1 Department of Epidemiology, University of Groningen, 10173 University Medical Center Groningen, Groningen, The Netherlands; 2 2865 Netherlands Interdisciplinary Demographic Institute, The Hague, The Netherlands; 3 Department of Sociology, Vrije Universiteit Amsterdam, Amsterdam, The Netherlands; 4 2873 Parnassia Psychiatric Institute, The Hague, The Netherlands; 5 Department of Psychiatry, University of Groningen, 10173 University Medical Center Groningen, Groningen, The Netherlands; 6 Department of Epidemiology, Mailman School of Public Health, Columbia University, New York, NY, USA

**Keywords:** childhood abuse, major depressive disorder, generalized anxiety disorder, body mass index, young adulthood

## Abstract

We examined whether childhood abuse is related to body mass index (BMI) in young adults and whether this relationship is mediated by depression and anxiety. Data are from the Dutch longitudinal cohort study TRAILS (n_females_ = 836, n_males_ = 719). At wave 4, childhood sexual, physical and verbal abuse, and lifetime major depressive disorder (MDD) and generalized anxiety disorder (GAD) were assessed. BMI was measured at wave 4 and 5 (mean age = 19.2/22.4 years). Sex-stratified structural equation models were estimated. Females who had experienced sexual abuse had a higher BMI at wave 4 (B = 0.97, 95%CI = [−0.01,1.96]) and a higher increase in BMI between wave 4 and 5 (B = 0.52, 95%CI = [0.04,1.01]) than females who had not experienced sexual abuse. Additionally, MDD and BMI at wave 4 were related in females (B = 1.35, 95%CI = [0.52,2.18]). MDD mediated the relationship between sexual abuse and BMI at wave 4 in females. In addition, sexual abuse moderated the relationship between MDD and BMI at wave 4. The relationship was stronger among females who had experienced sexual abuse than among females who had not. Prevention of BMI changes among females who experienced sexual abuse may thus be warranted, particularly when they developed MDD. MDD treatment, such as abuse-focused psychotherapy, may aid this prevention.

## Introduction

Childhood maltreatment, i.e., childhood sexual, physical and verbal abuse and neglect, has been associated with obesity in adulthood (Danese & Tan, 2014; Hemmingsson et al., 2014). In addition, childhood abuse has been related to cardiovascular disease and type 2 diabetes (Basu et al., 2017). Potential pathways via which childhood maltreatment could affect obesity and related conditions are alterations in health behaviors, biological factors, such as stress hormones, and mental health (Suglia et al., 2018). Depression is a mental health disorder that has been related to obesity (Mannan et al., 2016; Mühlig et al., 2016). While a relationship between childhood maltreatment and obesity in adulthood has been established, it is unclear when this relationship comes to expression. It could become apparent during the transition to adulthood (Schneiderman et al., 2015), as a result of individuals gaining autonomy over their health behaviors in this life period (Viner et al., 2015). Given that obesity is a risk factor for cardiovascular disease and type 2 diabetes, obesity could serve as an early marker for risk of cardiovascular disease and type 2 diabetes following childhood maltreatment (Suglia et al., 2018). Therefore, the first aim of this study was to investigate whether there is a relationship between childhood sexual, physical and verbal abuse and body mass index (BMI) in young adulthood.

A study into the possible mechanisms linking childhood abuse to adulthood obesity identified major depressive disorder (MDD) and generalized anxiety disorder (GAD) symptoms as possible mediator and suppressor, respectively, of the relationship between physical abuse and BMI (Francis et al., 2015). When examined in separate and sex-stratified models, only the suppressor effect of GAD symptoms was identified among females. GAD symptoms acted as a suppressor of the relationship, as there was a positive direct effect between physical abuse and BMI while the indirect effect via GAD symptoms was negative (MacKinnon et al., 2000). Another study found that childhood physical abuse, but not childhood sexual abuse, was positively related to BMI via depressive symptoms in females (Dedert et al., 2010). These results demonstrate several things. Firstly, MDD and GAD may mediate and suppress the relationship between childhood abuse and BMI. Secondly, childhood abuse may be linked to increases as well as decreases in BMI. Thirdly, mediation of the relationship between abuse and BMI may depend on the type of abuse studied. Finally, the relationships may be sex-specific. Therefore, the second aim of this study was to assess whether MDD and GAD mediated identified relationships between childhood sexual, physical, and verbal abuse and BMI among young adult females and males.

Childhood abuse may not only be related to changes in BMI via MDD and GAD, but may also serve as a moderator of the relationship between MDD/GAD and BMI (Salas et al., 2019; Stunkard et al., 2003). In individuals who experienced childhood abuse and who suffer from depression, alterations in biological reactions and in the body’s biology appear to be different than in individuals who suffer from depression, but experienced no childhood abuse (Danese et al., 2008; Heim et al., 2008a; Vythilingam et al., 2002). This suggests that the biology of depression is different according to whether individuals experienced childhood abuse or not (Heim et al., 2008b). It is shown that childhood abuse may influence the structure of the brain (McCrory et al., 2010). Depression and BMI are assumed to be related via unhealthy behaviors and/or biological mechanisms (Penninx, 2017). Biological mechanisms that are suggested to play a role in the relationship are systems that are involved in the stress response or that are influenced by the stress response, such as the autonomic nervous system, the HPA-axis and immuno-inflammatory reactions (Penninx, 2017). Given that depression and BMI may be related via biological mechanisms and the biology of depression may be influenced by childhood abuse, the relationship between MDD and BMI may also be different between individuals who experienced childhood abuse and individuals who did not (Penninx et al., 2013). Therefore, the third aim of this study was to examine moderation of the relationship between MDD/GAD and BMI by childhood abuse when mediation was examined.

## Methods

Data are from the TRacking Adolescents' Individual Lives Survey (TRAILS), a prospective cohort study of Dutch adolescents and young adults (Huisman et al., 2008; Oldehinkel et al., 2015). The TRAILS study was approved by the Central Committee on Research Involving Human Subjects (Dutch CCMO). From two municipalities in the North of the Netherlands children born between 1 October 1989 and 30 September 1990 were recruited and from three other municipalities in this area children born between 1 October 1990 and 30 September 1991 were recruited (n = 3483). Baseline data collection took place at schools. Written informed consent was obtained from parents and adolescents. Exclusion criteria were being in a primary school that did not agree to participate, having no parental or child consent, having a severe physical illness or mental retardation and not having a Dutch, Turkish or Moroccan speaking parent or parent surrogate (n = 548). At baseline, 2230 children were included (76.0% of eligible children in participating schools) with a mean age of 11 years. For the current study, data of wave 4 (October 2008 to September 2010) and wave 5 (April 2012 to November 2013) were used. Participants were aged between 18–21 years and between 21–23 years at these waves.

### Childhood Abuse

A questionnaire, developed by TRAILS, was used at wave 4 to collect information on sexual, physical and verbal abuse before the age of 16 years. Participants answered five questions on sexual and verbal abuse and six on physical abuse (see Supplementary Material, Table S1). Response options to questions on sexual abuse were ‘never’, ‘yes, once’ and ‘yes, more than once’. Sexual abuse was categorized into ‘no reported occurrence’ (0) and ‘one or more reported occurrences’ (1) due to the low frequency of reporting sexual abuse. Response options to questions on physical and verbal abuse ranged from ‘never’ (0) to ‘very often’ (4). For both types of abuse, the mean of the answers was taken for participants who provided answers to all questions concerning that abuse type. At wave 4, 1714 children filled out the questionnaire, 1644 participants provided complete information on sexual and verbal abuse and 1640 children provided complete information on physical abuse.

### Anthropometric Measurements

Trained research assistants performed anthropometric measurements at wave 4 and 5. Weight was measured with calibrated scales (Seca 876, Hamburg, Germany and Besthome EB813-SL) and height was measured with stadiometers/measuring tapes (Seca 201/222) with participants dressed in light clothes. BMI was calculated as weight divided by height squared (kg/m^2^). BMI measurements of 1574 participants were taken at wave 4 and of 1444 participants at wave 5.

### Major Depressive Disorder and Generalized Anxiety Disorder

At wave 4, the occurrence of major depressive disorder (MDD) and generalized anxiety disorder (GAD) was assessed with the Composite International Diagnostic Interview (CIDI) version 3.0 (Kessler & Üstün, 2004; Ormel et al., 2015). The CIDI version 3.0 is a structured diagnostic interview to assess mental disorders according to the fourth edition of the Diagnostic and Statistical Manual of Mental Disorders (DSM–IV, American Psychiatric Association, 2000). It was administered in person by a trained lay interviewer. Age of occurrence was also assessed. In the current study, participants were classified according to whether or not they had a lifetime MDD or GAD diagnosis. In total, 1584 participants participated in the CIDI.

### Covariates

Information on covariates was reported at wave 1. Parents reported on (1) mothers’ and (2) fathers’ education (in five categories from elementary to University education), (3) mothers’ and (4) fathers’ occupation (according to the International Standard Classification of Occupations (ISCO) (Ganzeboom & Treiman, 1996) and (5) household income. Scores on these indicators were standardized and averaged as a measure of socio-economic status (SES) (Amone-P’Olak et al., 2009). Parents also reported on their child’s ethnicity. Very few participants in the sample were non-Dutch (i.e., one or both parents born outside the Netherlands), therefore ethnicity was coded as either Dutch or non-Dutch. Age of the child was recorded at every measurement occasion.

### Statistical Analysis

**Figure 1.**
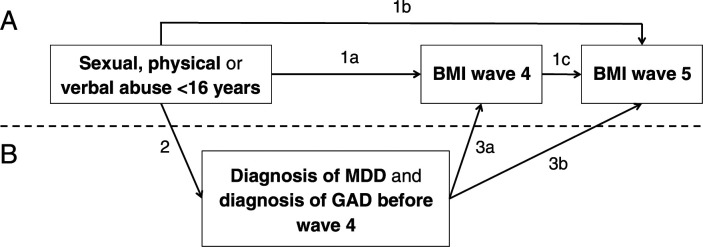
A. The studied relationship between abuse <16 years (assessed at wave 4, 18–21 years) and body mass index (BMI) at wave 4, wave 5 (21–23 years) and between wave 4 and wave 5. Abuse and BMI are regressed on parental socio-economic status (SES) and ethnicity. BMI at wave 4 is regressed on age at wave 4 and BMI at wave 5 is regressed on age at wave 5 and BMI at wave 4. Age at wave 5 is regressed on age at wave 4. B. Models including clinical diagnosis of MDD and GAD before wave 4 as mediators of the relationship between abuse and BMI at wave 4 and 5 are tested in a second step. MDD and GAD are additionally regressed on SES and ethnicity in these models.

Structural equation modeling was used. In the analysis, we included the variables in the temporal order in which they were measured and we expected them to have occurred (Figure 1). Childhood abuse that occurred before age 16 years was measured at wave 4. Lifetime MDD and GAD diagnosis were also measured at wave 4. BMI was measured at wave 4 (18–21 years) and wave 5 (21–23 years).

In a first step, the relationship between sexual, physical and verbal abuse and BMI at wave 4 and 5 was assessed using linear regression (Figure 1, paths 1a, 1b and 1c). This was done in separate models for each type of abuse. To adjust for possible confounding, paths from SES and ethnicity to the abuse and BMI variables and paths from age at wave 4 to BMI at wave 4, age at wave 5 to BMI at wave 5 and age at wave 4 to age at wave 5 were added.

In a second step, the associations between abuse and MDD/GAD and between MDD/GAD and BMI were tested using logistic and linear regression, respectively. Further, mediation of the identified associations between abuse and BMI by MDD and GAD was examined. MDD and GAD were added concurrently to the three models developed in the first step. To adjust for potential confounding, paths from SES and ethnicity to MDD and GAD were added. To assess mediation, we examined the indirect relationships between abuse and BMI via MDD and GAD. When testing indirect effects, we additionally examined whether the association between MDD/GAD and BMI was moderated by abuse experience. This was done as moderation of the relationship between MDD/GAD and BMI by childhood abuse can be expected and because indirect effect estimates may be incorrect in the presence of exposure-mediator interaction (Danese et al., 2008; Heim et al., 2008a; Valeri & VanderWeele, 2013; Vythilingam et al., 2002). To test moderation, BMI at wave 4 and 5 were additionally regressed on the interaction term of abuse and MDD/GAD.

Analyses were stratified according to sex, as sex differences have been identified in the relationship between depression and BMI and in mediation of the relationship between abuse and BMI by GAD (Anderson et al., 2006; Francis et al., 2015; Mannan et al., 2016; Mühlig et al., 2016; Richardson et al., 2003). As post-hoc analyses showed sex differences, stratification was justified.

As a sensitivity analysis, participants diagnosed with MDD and GAD before age 16 years only were excluded from the analyses. This was done as the temporal order of the experience of abuse and the diagnosis of MDD/GAD was unclear for these individuals. Please note that, whereas including the early onsets might lead to an overestimation of the associations under study, excluding them likely results in an underestimation. In a second sensitivity analysis, identified associations of variables with BMI at wave 4 were additionally adjusted for BMI at wave 1. This was done to examine to what extent associations of abuse and MDD/GAD with BMI at wave 4 may be overestimated because of preexisting differences in BMI at wave 1. However, this sensitivity analysis likely results in an underestimation of the associations under study as abuse and MDD/GAD may have occurred before wave 1 and have influenced BMI at wave 1. BMI at wave 1 was standardized based on age- and sex-specific reference curves of the International Obesity Task Force (IOTF), as BMI in childhood is dependent upon age and sex (Cole & Lobstein, 2012). BMI at wave 4 was regressed on abuse, BMI at wave 1, SES, ethnicity and age at wave 4. Additionally, paths from SES and ethnicity to abuse, from SES, ethnicity and age at wave 1 to BMI at wave 1 and from age at wave 1 to age at wave 4 were added. BMI at wave 5 was not incorporated in these models. MDD and GAD were added to these models in the same manner as in the main analysis.

Analyses were performed in MPlus version 7.3. Except for the mediation analysis, all analyses were performed using maximum likelihood estimation with robust standard errors (MLR). In these models, full information maximum likelihood was used to handle missing data. For the mediation analysis, weighted least squares (WLSMV) was used, as mediation by a binary variable cannot be tested using MLR. Standard errors of the indirect effect were obtained via bootstrapping (5000 iterations). As level of significance *p* < .05 was used.

## Results

The characteristics of the study sample (n_females_ = 836, n_males_ = 719) are described in Table 1.

**Table 1.** Descriptive statistics of the study sample.

	Females		Males	
	n (%)^a^	Mean (SD)	n (%)^a^	Mean (SD)
**Age wave 4 (years)**		19.17 (0.58)		19.23 (0.57)
**Age wave 5 (years)**		22.39 (0.60)		22.45 (0.59)
**Sexual abuse**	111 (13.7)		32 (4.7)	
**Physical abuse** ^b^	340 (42.1)	0.19 (0.35)	267 (39.6)	0.18 (0.33)
**Verbal abuse** ^b^	615 (75.8)	0.79 (0.80)	514 (76.3)	0.70 (0.69)
**Diagnosis MDD**	167 (20.1)		67 (9.4)	
**Diagnosis GAD**	43 (5.2)		13 (1.8)	
**BMI wave 4 (kg/m** ^**2**^ **)** ^c^		23.11 (3.99)		22.51 (3.75)
**BMI wave 5 (kg/m** ^**2**^ **)** ^c^		23.70 (4.33)		23.49 (3.68)
**Parental SES**		0.06 (0.76)		0.10 (0.78)
**Ethnicity**				
*Dutch*	744 (89.0)		646 (89.9)	
*Non-Dutch*	92 (11.0)		73 (10.2)	

MDD = major depressive disorder, GAD = generalized anxiety disorder, BMI = body mass index, SES = socio-economic status. The number of participants with valid data on a given variable ranges between 725-836 for females and 605-719 for males.

^a^Percentages shown are percentages of the number of participants with valid data on a given variable.

^b^The categories ‘1-2 times’ to ‘very often’ have been combined here to provide the number and percentage of individuals reporting an occurrence of physical and verbal abuse. In the analyses, physical and verbal abuse were included as continuous variables.

^c^Mean (SD) of the participants with BMI measurements at *both* wave 4 and 5: females wave 4 = 23.03 (3.87), females wave 5 = 23.71 (4.33), males wave 4 = 22.53 (3.72), males wave 5 = 23.49 (3.69).

### Childhood Abuse and BMI

Associations between childhood abuse and BMI are shown in Table 2 (see Figure 1(A) for a figure of the assessed associations).

**Table 2.** Direct, indirect and total associations between sexual, physical and verbal abuse and body mass index (BMI) at wave 4 and wave 5.

	Direct^a^				Indirect^b^		Total^c^	
	BMI wave 4		BMI wave 5		BMI wave 5		BMI wave 5	
	B	(95% CI)	B	(95% CI)	B	(95% CI)	B	(95% CI)
*Females*								
**Sexual abuse**	0.97	(−0.01–1.96)	0.52	(0.04–1.01)	0.93	(−0.02–1.88)	1.46	(0.36–2.55)
**Physical abuse**	0.08	(−0.72–0.88)	0.36	(−0.11–0.84)	0.08	(−0.70–0.85)	0.44	(−0.50–1.38)
**Verbal abuse**	0.06	(−0.31–0.42)	−0.14	(−0.34–0.05)	0.05	(−0.30–0.41)	−0.09	(−0.47–0.29)
*Males*								
**Sexual abuse**	0.79	(−0.81–2.39)	−0.27	(−0.96–0.41)	0.68	(−0.69–2.05)	0.41	(−0.97–1.78)
**Physical abuse**	−0.71	(−1.60–0.18)	0.33	(−0.10–0.76)	−0.61	(−1.38–0.16)	−0.28	(−1.19–0.64)
**Verbal abuse**	−0.14	(−0.56–0.29)	0.13	(−0.08–0.33)	−0.12	(−0.48–0.25)	0.01	(−0.41–0.43)

Females: n = 836, males: n = 719. BMI = body mass index. Sexual abuse is a dichotomized variable (no/yes). Associations are assessed in separate models per abuse type. All associations are adjusted for parental socio-economic status and ethnicity. Additionally, BMI at wave 4 and BMI at wave 5 are regressed on age at wave 4 and age at wave 5, respectively, and BMI at wave 5 is regressed on BMI at wave 4.

^a^The direct associations with BMI are path 1a (BMI wave 4) and path 1b (BMI wave 5) in Figure 1.

^b^This indirect association is the association between abuse and BMI at wave 5 through BMI at wave 4 (Figure 1, path 1a*1c).

^c^Total associations between abuse and BMI at wave 5 are equal to the sum of the direct and the indirect associations with BMI at wave 5.

In females, experience of sexual abuse versus no experience of sexual abuse was related to a higher BMI at wave 4 (Figure 1, path 1a: B = 0.97, 95%CI = [−0.01,1.96]), to a higher increase in BMI between wave 4 and wave 5 (Figure 1, path 1b: B = 0.52, 95%CI = [0.04,1.01]) and to a higher BMI at wave 5 (Figure 1, paths 1a*1c + 1b: B = 1.46, 95%CI = [0.36,2.55]). For females who experienced sexual abuse, the mean BMI at wave 4 was 23.96 (SD = 4.65) and the mean BMI at wave 5 was 24.95 (SD = 5.04). For females who did not experience sexual abuse, the mean BMI at wave 4 was 22.97 (SD = 3.78) and the mean BMI at wave 5 was 23.53 (SD = 4.20). There was no clear evidence for a relationship between physical abuse or verbal abuse and BMI at wave 4, wave 5 and between wave 4 and 5 in females.

In males, there was no clear evidence for any relationship between sexual, physical or verbal abuse and BMI at wave 4, wave 5 and between wave 4 and 5.

### Childhood Abuse and MDD/GAD

Associations between childhood abuse and MDD and GAD are shown in Table 3 (Figure 1, path 2).

**Table 3.** Direct associations between sexual, physical and verbal abuse and major depressive disorder (MDD) and generalized anxiety disorder (GAD) and between MDD and GAD and body mass index (BMI) at wave 4 and 5.

		Abuse and MDD/GAD^a^		MDD/GAD and BMI wave 4^b^		MDD/GAD and BMI wave 5^c^	
		B	(95%CI)	B	(95%CI)	B	(95%CI)
*Females*							
**Sexual abuse**	**MDD**	1.10	(0.66–1.54)	1.35	(0.52–2.18)	−0.09	(−0.50–0.32)
	**GAD**	1.29	(0.59–1.98)	−0.72	(−1.88–0.45)	0.21	(−0.90–1.33)
**Physical abuse**	**MDD**	0.79	(0.32–1.26)	1.46	(0.61–2.31)	−0.05	(−0.46–0.35)
	**GAD**	0.81	(0.26–1.36)	−0.57	(−1.71–0.56)	0.27	(−0.82–1.35)
**Verbal abuse**	**MDD**	0.53	(0.32–0.74)	1.47	(0.61–2.33)	0.02	(−0.39–0.42)
	**GAD**	0.75	(0.43–1.07)	−0.57	(−1.72–0.58)	0.41	(−0.69–1.51)
*Males*							
**Sexual abuse**	**MDD**	1.59	(0.77–2.40)	−0.34	(−1.36–0.69)	0.15	(−0.37–0.67)
	**GAD**	2.26	(0.94–3.57)	−1.10	(−2.84–0.65)	−0.63	(−1.74–0.48)
**Physical abuse**	**MDD**	1.06	(0.47–1.65)	−0.12	(−1.10–0.86)	0.07	(−0.45–0.60)
	**GAD**	1.00	(0.22–1.78)	−0.76	(−2.53–1.01)	−0.74	(−1.87–0.40)
**Verbal abuse**	**MDD**	0.88	(0.55–1.22)	−0.17	(−1.21–0.86)	0.06	(−0.49–0.61)
	**GAD**	0.37	(−0.55–1.29)	−0.84	(−2.63–0.94)	−0.69	(−1.84–0.46)

Females: n = 836, males: n = 719. MDD = major depressive disorder, GAD = generalized anxiety disorder, BMI = body mass index. Mediation of the relationship between abuse and BMI is assessed in separate models per abuse type (i.e. sexual, physical and verbal abuse), MDD and GAD are assessed concurrently in these models. Sexual abuse is a dichotomized variable (no/yes). All associations are adjusted for parental socio-economic status and ethnicity. Additionally, BMI at wave 4 and 5 are regressed on age at wave 4 and 5, respectively, and BMI at wave 5 is regressed on BMI at wave 4.

^a^Figure 1, path 2: examined using logistic regression, the coefficient describes change in log odds of MDD and GAD with every unit increase in abuse.

^b^Figure 1, path 3a.

^c^Figure 1, path 3b.

In females, all types of abuse were associated with increased odds of MDD and increased odds of GAD.

In males, all types of abuse were associated with increased odds of MDD and sexual and physical abuse were associated with increased odds of GAD. No statistically significant association was identified between verbal abuse and GAD in males (B = 0.37, 95%CI = [−0.55,1.29]).

### MDD/GAD and BMI

The estimates of the associations between MDD/GAD and BMI at wave 4 and 5, as identified in the three different models including the three different types of abuse, can be found in Table 3 (Figure 1, path 3a and path 3b).

There was a relationship between MDD and BMI at wave 4 in females (B = 1.35–1.47, 95%CI = [0.52, 2.18–0.61, 2.33]). There was no clear evidence for a direct association between MDD and BMI at wave 5 or for an association between GAD and BMI at wave 4 or 5.

In males, no statistically significant associations between MDD and GAD and BMI at wave 4 or 5 were identified.

### Mediation and Moderation Analyses

We limited our mediation analysis to the relationship between sexual abuse and BMI among females. We found evidence for an indirect relationship between sexual abuse and BMI at wave 4 via MDD (*p* = .015). However, we also found evidence for moderation of the relationship between MDD and BMI at wave 4 by sexual abuse in females. The relationship was stronger among females who had experienced sexual abuse (n = 111, B = 3.40, 95%CI = [1.57, 5.22]), than among females who had not (n = 698, B = 0.62, 95%CI = [−0.27, 1.51]).

There was no clear evidence for an indirect relationship between sexual abuse and BMI at wave 5 via MDD or GAD – other than via BMI at wave 4. There was also no clear evidence for moderation of the direct relationships between MDD/GAD and BMI at wave 5 by sexual abuse.

### Covariates

The associations of the covariates with childhood abuse, MDD and GAD and BMI were slightly different in the different models, i.e., the models including the three different types of abuse and the models with and without MDD and GAD.

Among females, non-Dutch ethnicity seemed associated with a higher odds of sexual abuse and MDD, and with higher physical abuse and verbal abuse compared with Dutch ethnicity. However, the standard errors of the associations with sexual abuse and MDD were large and the associations were not statistically significant in all different models. Among females, SES was negatively associated with both verbal abuse and BMI at wave 4.

Among males, non-Dutch ethnicity compared with Dutch ethnicity was associated with a higher odds of sexual abuse, higher verbal abuse and lower BMI at wave 4. The effect estimate of the direct association of ethnicity with BMI at wave 5 was also indicative of negative association when comparing non-Dutch ethnicity to Dutch ethnicity, but the standard error was large and the association was not statistically significant in all models. Non-Dutch ethnicity also seemed associated with higher physical abuse compared with Dutch ethnicity, but the association was not statistically significant. Among males, SES was negatively associated with physical abuse and BMI at wave 4, although the effect estimate of the association with physical abuse was small. The effect estimate of the direct association between SES and BMI at wave 5 was also negative, but the association was not statistically significant. SES further seemed associated with lower odds of sexual abuse, but the association was not statistically significant.

### Sensitivity Analysis

In the first sensitivity analysis, participants with a diagnosis of MDD and GAD before age 16 years, but not after age 16 years, were excluded. Therefore, the sample size for this analysis was smaller (n_females_ = 787, n_males_ = 705). Differences with the main analysis were that the association between sexual abuse and BMI at wave 4 in females was attenuated (B = 0.62, 95%CI = [−0.31,1.56]). Further, the relationship between physical abuse and GAD in males was attenuated (B = 0.75, 95%CI = [−0.10,1.59]). Evidence for an indirect relationship between sexual abuse and BMI at wave 4 via MDD in females also became weaker (*p* < .10). Unlike in the main analysis, we found evidence for moderation of the relationships between GAD and BMI at both wave 4 and 5 by sexual abuse among females. We identified a negative association between GAD and BMI at wave 4 among females who had experienced sexual abuse (n = 95, B = −2.15, 95%CI = [−4.12,−0.19]), but not among females who had not experienced sexual abuse (n = 667, B = 0.67, 95%CI = [−1.05,2.39]). This same pattern was visible for the association between GAD and BMI at wave 5, although the association was significant neither among females who had experienced sexual abuse (n = 95, B = −1.25, 95%CI = [−3.00,0.50]) nor among females who had not (n = 667, B = 1.04, 95%CI = [−0.62,2.69]).

In the second sensitivity analysis, identified associations with BMI at wave 4 were additionally adjusted for BMI at wave 1. Sample sizes were larger than in the main analysis as individuals who participated at wave 1, but not at wave 4, were included in these analyses. Differences with the main analysis were that the association between sexual abuse and BMI at wave 4 in females was attenuated (n = 1106, B = 0.48, 95%CI = [−0.20,1.17]). In addition, the effect estimate of the association between MDD and BMI at wave 4 was about half the size as in the main analysis (n = 1106, B = 0.66, 95%CI = [0.06,1.27]). Evidence for moderation of the relationship between MDD and BMI at wave 4 by sexual abuse also became weaker (*p* = .04). However, as in the main analysis, the relationship was stronger among females who had experienced sexual abuse (n = 118, B = 1.82, 95%CI = [0.60,3.04]), than among females who had not (n = 771, B = 0.21, 95%CI = [−0.47,0.88]).

## Discussion

In this study, we examined the relationship between childhood abuse and BMI in young adulthood, and mediation of this relationship by major depressive disorder (MDD) and generalized anxiety disorder (GAD). Of the three types of abuse we distinguished (i.e. sexual, physical and verbal abuse), only sexual abuse in females was related to higher BMI at the end of adolescence and a higher increase in BMI in young adulthood. MDD mediated the relationship between sexual abuse and BMI at the end of adolescence in females. Sexual abuse also moderated the relationship between MDD and BMI at the end of adolescence. The relationship between MDD and BMI at the end of adolescence was particularly present among females who experienced sexual abuse.

### Childhood Abuse and Body Mass Index

The associations between sexual abuse and BMI in females are in line with a study identifying higher increases in BMI between childhood and young adulthood among females who did experience sexual abuse as opposed to females who did not (Noll et al., 2007). The transition to adulthood seems to be a crucial period for the emergence of changes in BMI development following sexual abuse in females. No statistically significant associations were identified between sexual abuse and BMI in males, but this could be due to a lack of power. Further, we found no evidence for an association between physical and verbal abuse and BMI in females or males. This suggests that the relationships between both physical and verbal abuse and BMI, which are identified in adults (Danese & Tan, 2014; Hemmingsson et al., 2014), have not come to expression yet in young adulthood.

### Childhood Abuse and Major Depressive Disorder and Generalized Anxiety Disorder

In line with earlier studies, strong associations were found between childhood abuse and diagnosis of MDD and GAD (Fernandes & Osório, 2015; Infurna et al., 2016; Li et al., 2016; Lindert et al., 2014; Mandelli et al., 2015; Norman et al., 2012). No statistically significant association was identified between verbal abuse and GAD in males. It is possible that males do not become anxious, or at least do not develop GAD, following verbal abuse (Fernandes & Osório, 2015). It is also possible that the association is simply not identified due to a lack of power.

### Major Depressive Disorder and Generalized Anxiety Disorder and Body Mass Index

Associations were identified between diagnosis of MDD and BMI at the end of adolescence in females. Interestingly, this association was not identified in males. Several studies found a stronger relationship between depression and subsequent obesity for females than males (Korczak et al., 2013; Mannan et al., 2016; Mühlig et al., 2016). Possibly, females with MDD are more prone than males with MDD to display unhealthy behaviors (Camilleri et al., 2014). In addition, differences between males and females could be the result of biological differences (Mannan et al., 2016).

No statistically significant associations were found in this study between GAD and BMI. Previously, a meta-analysis revealed moderate evidence for a positive cross-sectional association between anxiety disorders and obesity (Gariepy et al., 2010). However, none of the included studies assessed the relationship between GAD and BMI in young adulthood. A study at the end of young adulthood identified a negative association between GAD and BMI (Francis et al., 2015). Results of the limited number of studies into the relationship thus point into different directions, while studies are difficult to compare due to between study differences.

### Mediation and Moderation Analyses

We identified mediation of the relationship between sexual abuse and BMI at the end of adolescence by diagnosis of MDD in females. Previous research identified mediation of the relationship between childhood physical abuse and BMI by MDD symptoms and identified GAD symptoms as a suppressor (Francis et al., 2015). Differences in study results could be caused by the fact that in the previous study participants were recorded cases of abuse and MDD and GAD symptoms were measured instead of MDD and GAD diagnoses. However, both our and the previous study suggest that a higher BMI in individuals who experienced abuse compared to individuals who did not experience abuse may, partially, be prevented by preventing MDD development or by preventing BMI gain in those who developed MDD.

We also found evidence for moderation of the relationship between MDD and BMI by sexual abuse in females. Possibly, MDD and BMI are more strongly related in individuals who experienced childhood abuse as biological alterations in response to childhood abuse are at the root of both conditions. This idea is in line with research showing biological differences between depressed patients who did experience childhood abuse and depressed patients who did not (Danese et al., 2008; Heim et al., 2008a; Vythilingam et al., 2002). In addition, studies have shown that the clinical course of and treatment success in MDD is influenced by adverse childhood experiences (Heim et al., 2004; Nanni et al., 2012; Nelson et al., 2017). This study also suggests that MDD treatment needs to be informed by childhood sexual abuse.

### Prevention and Intervention Efforts

Successful prevention and intervention efforts for this population likely need to be multifaceted (Britto et al., 2017). Prevention of childhood abuse is most effective if it starts early in life (Britto et al., 2017). Prevention programs focusing on parenting and caregiving and led by professionals visiting the home seem to hold promise in this regard (Britto et al., 2017). Parenting programs, focusing on enhancing knowledge about parenting, building parenting skills, enhancing competency and parent support, seem to be successful as a primary, secondary and tertiary prevention of childhood abuse (Chen & Chan, 2016). When considering interventions to reduce the adverse consequences of childhood abuse, it seems crucial that MDD treatment in individuals who experienced adverse childhood experiences includes psychotherapy (Heim et al., 2004; Nemeroff et al., 2003). Abuse-focused psychotherapy may reduce depression among adults who experienced childhood sexual abuse (Martsolf & Draucker, 2005) and trauma-focused cognitive behavioral therapy seems promising when it comes to reducing MDD symptoms in preschool age children who experienced trauma (Cummings et al., 2012).

### Covariates

The results generally suggest an association between non-Dutch ethnicity and higher odds of sexual abuse and higher physical and verbal abuse among both females and males, and some negative associations between SES and abuse experience. However, both ethnicity and SES were generally not related to MDD and GAD, and mainly SES was associated with lower BMI. This suggests that individuals from non-Dutch ethnicity generally experience more abuse, or are more likely to report abuse, while they are not more likely to experience MDD and GAD, or have a higher BMI, independent of abuse experience. It is important to emphasize that this cohort is from the North of the Netherlands where a relatively small share of the population is of non-Dutch ethnicity. We would have potentially identified more differences if we had been able to make comparisons between specific ethnic groups. In this study, we did not examine whether the associations under study differ between individuals of Dutch and non-Dutch ethnicity. If the associations under study are the same for individuals from different ethnic groups, the suggested prevention efforts would, at a minimum, likely need to be culturally sensitive, and take into account the potentially different context in which the abuse occurred (Martsolf & Draucker, 2005).

### Strengths and Limitations

A strength of this study is the use of longitudinal data. We used detailed information on the occurrence of abuse before age 16, MDD and GAD before the end of adolescence and BMI at the end of adolescence and in young adulthood, allowing us to test a temporal relationship between these variables. Moreover, diagnosis of MDD/GAD was assessed with a structured diagnostic interview and BMI was determined using objective height and weight measurements obtained by trained research assistants. Finally, we assessed moderation of the relationship between MDD and BMI by childhood sexual abuse. This highlighted that there is indeed moderation of the relationship, which may hold important implications from a prevention and intervention perspective.

A limitation of the current study is that participants reported childhood abuse retrospectively using a self-report questionnaire. However, the alternative of examining official cases of childhood abuse carries the downside of only including the subset of abuse cases that comes to professional attention (Gilbert et al., 2009). Another limitation is that the questionnaire used was developed by TRAILS. This was done, because none of the existing questionnaires at the time was considered fully appropriate for use in the TRAILS sample in terms of item content or number of items. A third limitation is that we did not adjust for all potential confounders of the relationships under study. However, adjusting for biological factors, alcohol and drug abuse, and other mental health conditions, would have likely resulted in overadjustment of the relationships in this study (Colman et al., 2012; Penninx, 2017). A fourth limitation is that we could not specifically adjust for BMI before the occurrence of MDD. However, when adjusting the association between MDD diagnosis and BMI at the end of adolescence for early adolescent BMI, a relationship between MDD diagnosis and BMI at the end of adolescence was still identified. However, we cannot be sure that MDD is affecting BMI. There likely is a reciprocal relationship between MDD and BMI and there also may be third factors – that could be a consequence of childhood abuse – that are able to influence MDD and GAD occurrence and BMI, such as the potential confounders mentioned above (MacKinnon et al., 2007). This requires us to be cautious in our interpretation of the results. The association between MDD and BMI may partly be due to BMI affecting MDD, or third factors affecting both conditions. Another limitation is the non-random nonresponse at the fourth wave of TRAILS (Ormel et al., 2015, 2017). For example, nonresponders at wave 4 more often had low socio-economic status compared to responders (Nederhof et al., 2012). Finally, the fact that the CIDI is to be applied by trained lay interviewers instead of clinical professionals could be a limitation (Ormel et al., 2015).

## Conclusion

In this study, a relationship was identified between sexual abuse and BMI among females, specifically in young adulthood. This implies that young adulthood is a crucial life phase for the development of obesity after the experience of sexual abuse in females, and that, when prevention of sexual abuse has failed, interventions to prevent obesity development following sexual abuse should be planned before the end of adolescence. Sexual abuse in males and other forms of abuse in females and males were not related to BMI this early in adulthood. In contrast, the occurrence of MDD and GAD in individuals who experienced childhood abuse was already elevated before the end of adolescence. As MDD and BMI in females are related, especially among females who experienced childhood sexual abuse, prevention of MDD and – tailored – MDD treatment could additionally carry physical health benefits.

## Supplemental Material

Supplemental Material - Relationship Between Childhood Abuse and Body Mass Index in Young Adulthood: Mediated by Depression and Anxiety? Click here for additional data file. Supplemental Material for Relationship Between Childhood Abuse and Body Mass Index in Young Adulthood: Mediated by Depression and Anxiety? by Leonie K. Elsenburg, Aart C. Liefbroer, Annelies E. Van Eeden, Hans W. Hoek, Albertine J. Oldehinkel, and Nynke Smidt in Child Maltreatment
