# Supplementary material for: Relationship Between Childhood Abuse and Body Mass Index in Young Adulthood: Mediated by Depression and Anxiety?
Source: Child Maltreat. 2022 Jun 2;28(2):286–96. doi: 10.1177/10775595221092946 (PMC10028135; doi:10.1177/10775595221092946)
Supplement: Supplemental Material - Relationship Between Childhood Abuse and Body Mass Index in Young Adulthood: Mediated by Depression and Anxiety? [file sj-pdf-1-cmx-10.1177_10775595221092946.pdf]

Supplementary Material

Table S1. Questions used to assess sexual (SA), physical (PA) and verbal abuse (VA)

**Questions**	**Type of abuse**
Before you were 16 years, did an adult family member, acquaintance of the family or a stranger ever	
1. Show you his/her genitals, or masturbate in front of you?	SA
2. Touch you in a sexual manner?	SA
3. Make you or force you to touch him/her in a sexual manner?	SA
4. Attempt to have any kind of intercourse (oral, vaginal, anal) with you?	SA
5. Have a kind of intercourse (oral, vaginal, anal) with you?	SA
Below is a list of ways in which your father or mother can respond. Indicate how often they displayed these responses before you were 16 years. Questions also apply to foster and stepparents.	
6. My mother and/or father shouted, screamed or yelled at me	VA
7. My mother and/or father hit me with a belt, brush, stick or other hard object	PA
8. My mother and/or father hit me with her/his fist or kicked me (very) hard	PA
9. My mother and/or father hit me with her/his bare hand	PA
10. My mother and/or father threatened to hit me, but did not do it	VA
11. My mother and/or father called me stupid or lazy or something similar	VA
12. My mother and/or father said that I would be sent away or that I would have to leave the house	VA
13. My mother and/or father has forcefully shaken or pinched me	PA
14. My mother and/or father has beaten me up (i.e. hit very hard in succession)	PA
15. My mother and/or father has cursed at me or called me names	VA
16. My mother and/or father has threatened me with a knife or other weapon	PA

Questions were translated for publication. Answer possibilities of questions 1-5 were ‘No, never’, ‘Yes, once before the age of 16 years’ and ‘Yes, more than once before the age of 16 years’. Answer possibilities of questions 6-16 were ‘Never’, ‘1-2 times’, ‘Sometimes’, ‘Often’ and ‘Very often’.
